# Colostrum replacer and bovine leukaemia virus sero-positivity

**DOI:** 10.1186/1742-4690-11-S1-P59

**Published:** 2014-01-07

**Authors:** Bhudipa Choudhury, Christopher Finnegan, Jean-Pierre Frossard, Chris Venables, Falko Steinbach

**Affiliations:** 1 Department of Virology, AHVLA, Weybridge, Surrey, UK

## 

Bovine Leukemia Virus (BLV), a Deltaretrovirus in the *Retroviridae* family, is the causative agent of Enzootic Bovine Leucosis (EBL). Whilst EBL is still endemic in the Americas and Eastern Europe, most Western European countries are disease free in accordance with European Union legislation e.g. Great Britain has held EBL-free status since 1999. BLV infection is life-long and the presence of antibody and integrated proviral DNA are indicators of exposure to the virus.Screening for antibodies is the primary means of diagnosis. Here we present three case reports of animals which tested BLV antibody positive. As there was no evidence that the animals or their in-contacts were infected with BLV the result was somewhat confusing. Investigation revealed that the animals had been fed a colostrum replacer that was produced outside of Great Britain. As BLV is endemic in some countries, it was considered that BLV antibodies may have been present in the colostrum replacer and thus passively acquired resulting in BLV sero-positivity. The hypothesis was supported by the declining serological antibody titre seen on serial blood sampling. We present data on the serological and molecular testing conducted on the animals and product in support of the hypothesis. The above mentioned cases were all linked by the same brand of colostrum replacer; in addition we provide data derived from the investigation of colostrum replacers from other manufacturers. The policy and International Trade implications will also be discussed.

